# Trends in research of exosomes associated with breast cancer over the past decade: a scientometric analysis

**DOI:** 10.3389/fonc.2023.1273555

**Published:** 2023-10-03

**Authors:** Jiaxin Wu, Daitian Zheng, Haiting Wang, Zhongming Zhou, Qiuping Yang, Jinyao Wu, Huiting Tian, Zeqi Ji, Lingzhi Chen, Jiehui Cai, Yexi Chen, Zhiyang Li

**Affiliations:** Department of Thyroid, Breast and Hernia Surgery, General Surgery, The Second Affiliated Hospital of Shantou University Medical College, Shantou, Guangdong, China

**Keywords:** breast cancer, exosomes, scientometrics, Bibliometrix, VOSviewer, CiteSpace

## Abstract

**Introduction:**

Breast cancer remains a significant global health challenge, accounting for 2.3 million new cases in 2020 and ranking as the most prevalent cancer by incidence and the fourth in cancer-related mortality worldwide. In China, breast cancer also rapidly increases incidence and burden. The research of exosomes in breast cancer has attracted more and more attention and has a rapid development. Recognizing the pivotal role of exosomes in breast cancer research, we have undertaken a comprehensive scientometric analysis of pertinent scholarly articles published over the past decade to elucidate the current research landscape for researchers.

**Methods:**

In this study, we gathered all pertinent publications from the Web of Science. Biblioshiny (a web interface for Bibliometrix), VOSviewer software, and CiteSpace software were used to analyze the information on publications, including global trends, countries, institutions, journals, authors, keywords, and citations.

**Results:**

A total of 1,239 articles and 625 review articles were retrieved. The annual global publication output has an increased trend in recent decades overall. China contributed the most articles. The publications of the USA had the most total link strength. Nanjing Medical University had the most total link strength. The most relevant source was the *International Journal of Molecular Sciences*. Tang JH contributed the most articles and had the highest H-index, G-index, and total link strength. The most cited document was “Tumor exosome integrins determine organotropic metastasis”, with 2730 citations. The basic themes included “exosomes”, “expression”, “cells”, “identification”, “biomarkers”, and “serum”. The keyword “membrane vesicle” had the strongest bursts. The keywords “target”, “biology”, “suppressor cell”, “molecular mechanism”, “tumor progression”, “inhibitor”, and “model” appeared as prominent focal points in current research and active areas of exploration.

**Conclusion:**

Over the past decade, exosome research in breast cancer has undergone a discernible evolution, shifting from broader investigations of exosome roles to focused exploration of specific pathways relevant to breast cancer. Notably, the emphasis has extended to the clinical application of exosomes as biomarkers and potential therapeutic agents in breast cancer treatment.

## 1 Introduction

Breast cancer remains a significant global health challenge, accounting for 2.3 million new cases in 2020 and ranking as the most prevalent cancer by incidence (1). Furthermore, breast cancer has the fourth mortality following lung, colorectal, liver, and stomach (1). Simultaneously, in China, breast cancer also has a rapid increase in incidence and burden (2). The current clinical methods of diagnosis and therapy have some limitations (3). Given the escalating burden of breast cancer, the imperative to identify novel and efficacious therapeutic interventions that can address this global challenge has become paramount.

Exosomes, extracellular vesicles responsible for intercellular communication, are produced by both normal and malignant cells (4, 5). Within these tumor-derived exosomes, genetic information, particularly microRNAs, has been found to exert significant influence on the proliferation and invasion of various tumors, including breast cancer, by modulating gene expression in fibroblasts or epithelial cells (6, 7). Exosomes promote the growth of tumor cells, regulate tumor metastasis, aid tumor cells in evading immune surveillance, and can modulate the activity of immune cells (8–16). Growing evidence shows that exosomes of tumor cells also mediate chemoresistance (9, 17–19). Given the abundance and tumor-specific characteristics of exosomes, they hold potential as promising candidates for breast cancer diagnosis and therapeutic biomarkers upon isolation and purification (8, 9, 13, 20–22). Exosomes have not been used widely in clinical practice though related researches are in full swing.

Given the crucial significance of exosomes and the dynamic nature of research in this field, it becomes imperative to thoroughly investigate the current state of relevant studies and anticipate future trends. In light of this urgency, we have undertaken a scientometric analysis of pertinent articles to elucidate the present research landscape, with a specific focus on exosomes in the context of breast cancer. Our study comprehensively examines publications from the past decade, with the aim of identifying leading countries and authors in terms of research output, discerning influential journals, spotlighting prevailing hot topics, and offering insights into prospective research directions for the scientific community.

## 2 Methods

### 2.1 Data collection

**Figure 1:**
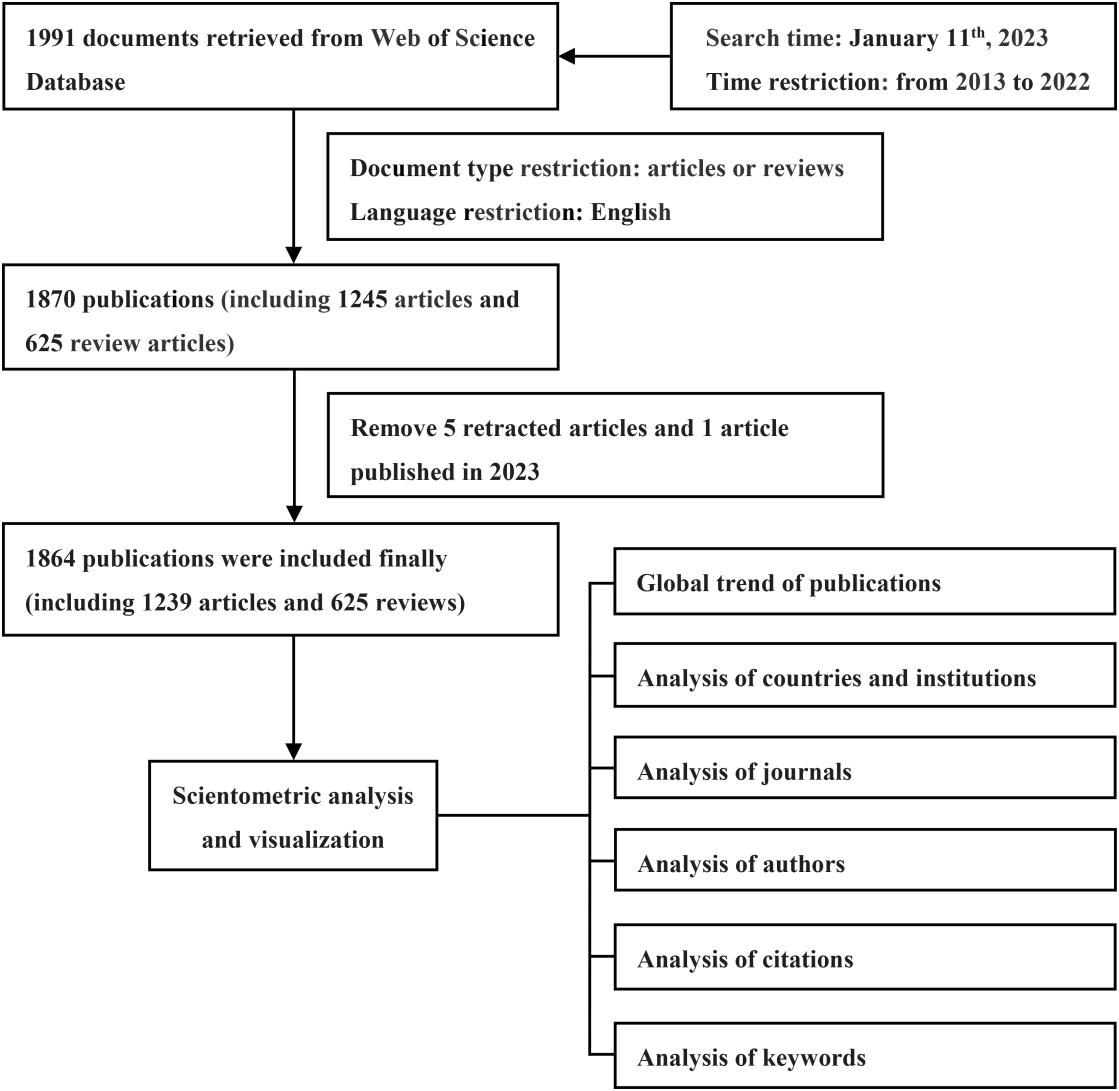
Flowchart of data collection and analysis.

All pertinent data concerning the correlation between exosomes and breast cancer were extracted from the Web of Science (WoS) Core Collection on January 11^th^, 2023. The search encompassed various databases, including ESCI (2017–present), SSCI (2003–present), SCI-EXPANDED (2003–present), A&HCI (2003–present), IC (1993–present), and CCR-EXPANDED (1985–present). Medical subject headings (Mesh) and entry terms “exosome” and “breast cancer” were employed as search strategies. The search query was structured as follows: #1, TS=(“exosome*”); #2, TS=(“breast cancer*”) OR TS=(“breast neoplasm*”) OR TS=(“breast carcinoma*”) OR TS=(“mammary neoplasm*”) OR TS=(“mammary carcinoma*”) OR TS=(“mammary cancer*”) OR TS=(“breast tumor*”); #3, “#1” and “#2”. The publications under analysis were limited to the timespan of 2013 to 2022, resulting in a total of 1,991 documents. For meticulous selection, the language was restricted to English, while document type criteria were set to include articles and reviews. After thorough scrutiny, the dataset comprised 1,870 documents, encompassing 1,245 articles and 625 review articles. A meticulous exclusion process was carried out, dismissing 121 publications based on specific criteria, including 100 meeting abstracts, five corrections, ten editorial materials, two letters, one retraction, one non-English review article, and two non-English articles. In addition, five more retracted publications and one article published in 2023 were found and excluded after using the Zotero software. In conclusion, the number of publications was 1,864, including 1,239 articles (constituting 66.47% of the total), and 625 review articles (33.53%). A visual representation of the step-by-step literature collection and analysis process is provided in **Figure 1**.

### 2.2 Data analysis

The data extracted from the chosen publications were subjected to analysis using VOSviewer software, Biblioshiny (a web interface for Bibliometrix), and CiteSpace software, enabling an automated examination of crucial aspects, such as countries, institutions, journals, authors, keywords, and citations.

For the comprehensive scientometric analysis, we utilized Bibliometrix, an R-tool available on RStudio (version 4.2.1), enabling the generation of visual representations of the results (23). Upon importing the raw data files into the Biblioshiny website, we gained a comprehensive overview of the documents, encompassing essential details such as the timespan, the number of sources, the number of documents, authors, document contents, document types, and the number of references. With this information, we performed a preliminary assessment to ensure compliance with inclusive criteria. Subsequently, we examined additional key aspects, including annual scientific production, countries and institutions’ contribution, the most relevant journals, authors (most relevant authors, the cited number of authors’ articles, author impact ranked by H-index, G-index, and M-index), and keywords. Additionally, the co-occurrence network of keywords proved instrumental in identifying prevailing areas of research interest.

We employed VOSviewer (version 1.6.18) as a powerful tool for conducting a comprehensive analysis of the most cited documents. Additionally, VOSviewer facilitated the construction of co-citation network analyses for references and network analyses to identify keyword co-occurrence patterns. Moreover, VOSviewer enabled the assessment of link strength across various entities, including authors, institutions, and countries (24).

CiteSpace was applied to discern keywords exhibiting robust citation bursts (25).

## 3 Results

### 3.1 Global trend of publications

**Figure 2:**
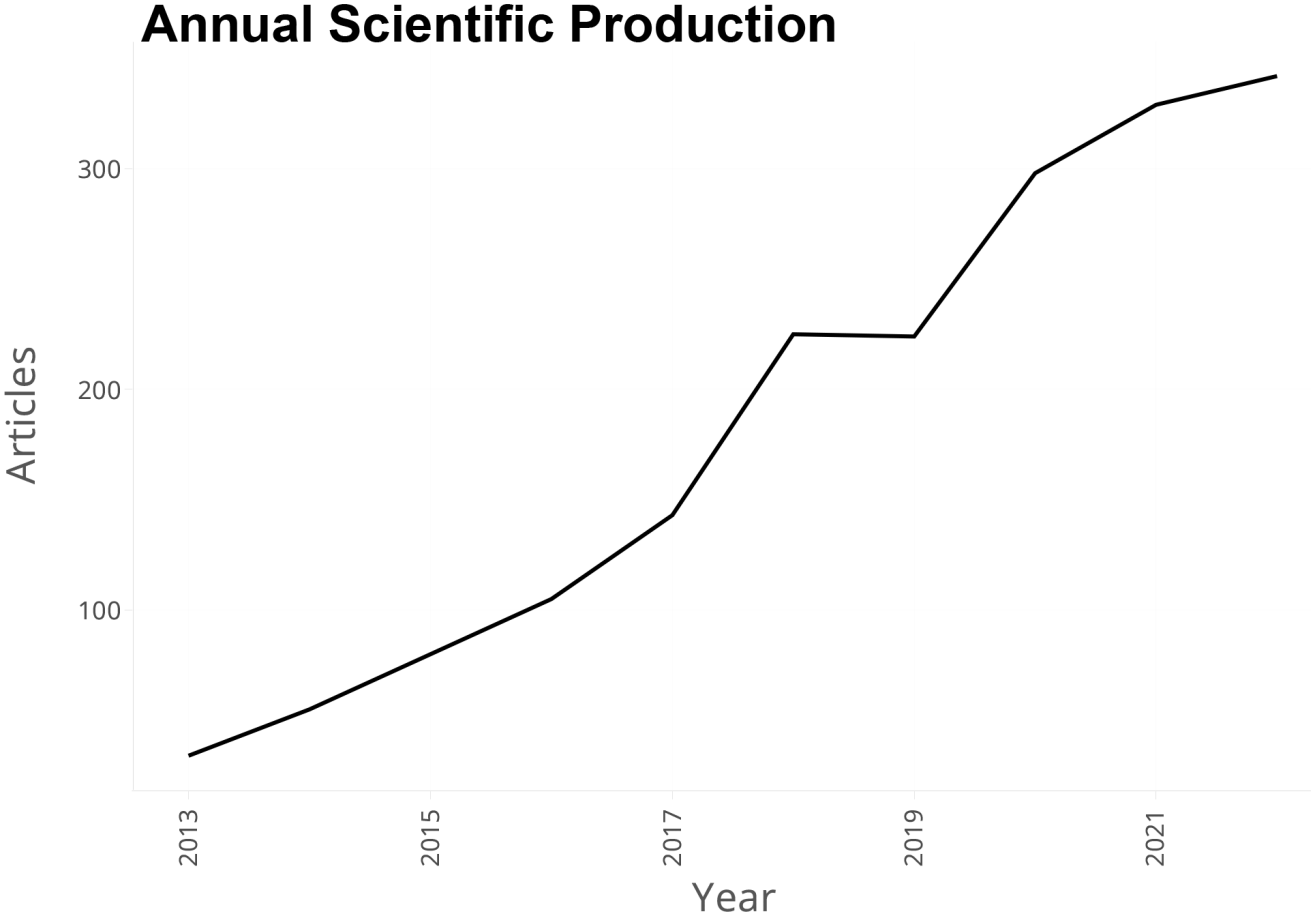
Annual scientific production from 2013 to 2022.

One thousand eight hundred sixty-four articles related to breast cancer and exosomes from 2013 and 2022 were retrieved from WoS. The minimum number was 34 in 2013. The maximum is 342 in 2022. And strong upward trend was exhibited from 2013 and 2022, except for 2018 (n=225) to 2019 (n=224), in which the number of publications declined a little (**Figure 2**). The annual growth rate is 29.24%. It is worth noting that the growth rate reached 57.34%, and the number of publications broke through 200 in 2018.

### 3.2 Analysis of countries and institutions

The dissemination of publications extended to 59 countries and regions. A blue-coded world map was made to analyze global contributions to breast cancer and exosome research (**Supplementary Figure S1**). In these countries, China contributed the most articles (n=645, 34.6% of total articles), followed by the USA (345, 18.5%), Italy (97, 5.2%), and Iran (80, 4.3%) (**Table 1**). The USA claimed the most substantial number of cited publications (n=26955), followed by China (n=21342), Japan (n=3888), and Italy (n=3375) (**Supplementary Table S1**).

**Table 1:** Top 10 countries with the largest number of articles.

Country	Articles	SCP	MCP	Freq	MCP_Ratio
China	645	561	84	0.346	0.130
USA	345	251	94	0.185	0.272
Italy	97	67	30	0.052	0.309
Iran	80	61	19	0.043	0.237
Germany	73	51	22	0.039	0.301
Korea	72	58	14	0.039	0.194
Japan	61	50	11	0.033	0.180
India	49	27	22	0.026	0.449
Australia	39	23	16	0.021	0.410
Spain	36	24	12	0.019	0.333

SCP, Single collaboration publication; MCP, Multiple collaboration publication; Freq, Frequency.

**Figure 3:**
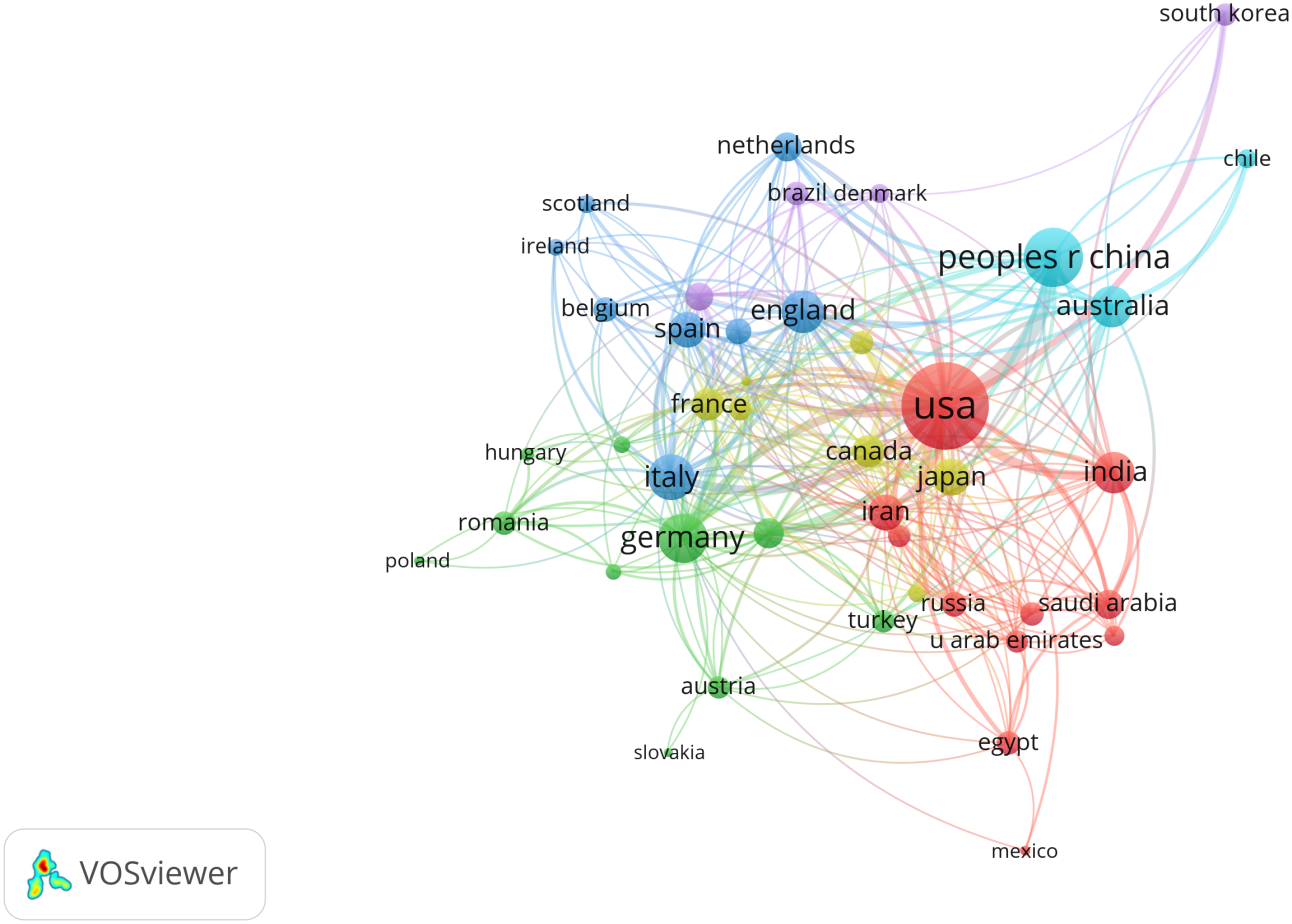
Network map of co-authorship between countries with more than five publications.

The co-authorship analysis involved a comprehensive examination of 44 countries that surpassed the threshold of five publications in the field (**Figure 3**). In these countries, the publications of the USA had the most total link strength (348), China ranked second (150), followed by Germany (104) and Italy (87).

In this domain, 2,211 institutions participated in contributing to the body of research. Nanjing Medical University contributed the most articles (n=201, 10.78%), followed by Fudan University (n=88, 4.72%), the University of Texas MD Anderson Cancer Center (n=77, 4.13%), Shahid Beheshti University of Medical Sciences (n=57, 3.06%) (**Supplementary Table S2**).

**Figure 4:**
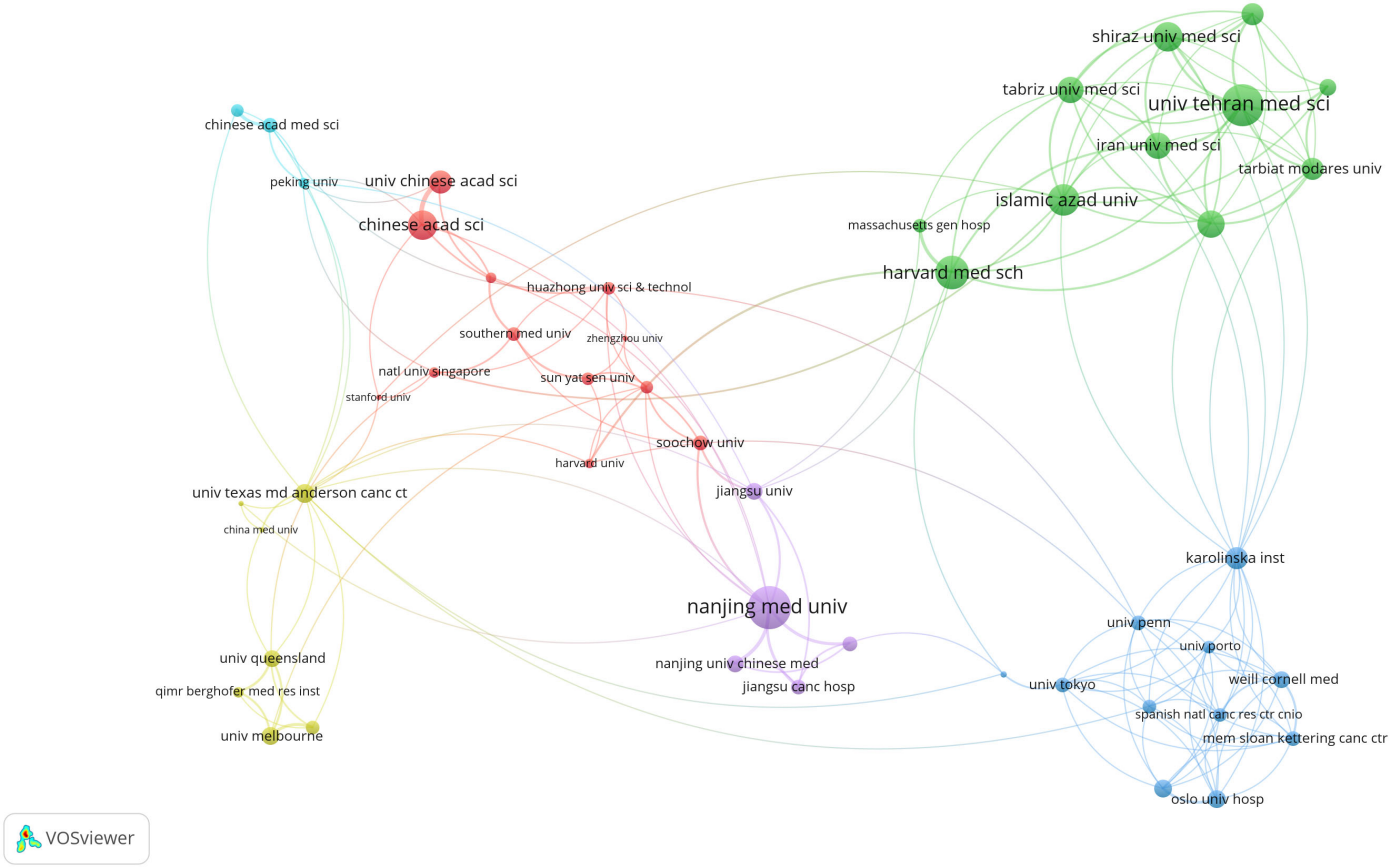
Network map of co-authorship between institutions with more than five publications. The distance between nodes indicates the strength of the relationship.

Subsequently, a detailed examination of co-authorship among organizations, featuring more than five publications, yielded a dataset comprising 204 organizations. Remarkably, Nanjing Medical University emerged as the institution with the most robust link strength (n=62), followed by Harvard Medical School (n=48) and the Chinese Academy of Sciences (n=41) (**Figure 4**).

### 3.3 Analysis of journals

The related articles were published in a total of 548 journals. The top relevant source was the *International Journal of Molecular Sciences* (number of the related articles=70, 3.76% of total articles), followed by *Cancers* (64, 3.43%), *Frontiers in Oncology* (46, 2.47%), and *Scientific Reports* (44, 2.36%) (**Supplementary Table S3**).

**Figure 5:**
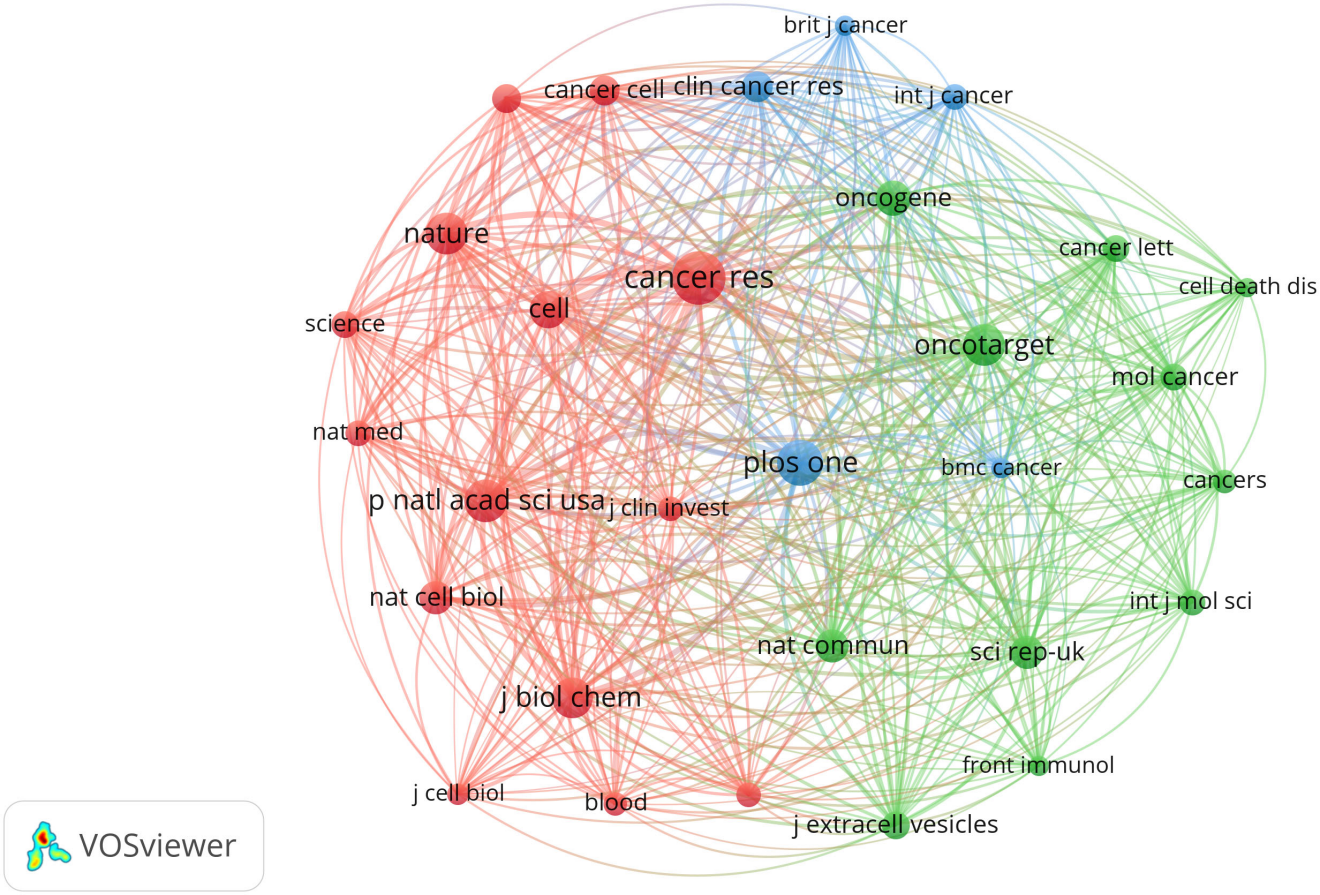
Network map of journals that were co-cited in more than 60 publications.

We examined a total of 415 journals encompassing all publications that garnered co-citations exceeding the threshold of 60 citations (**Figure 5**). *Cancer Research* had the most link strength (n=501423), followed by *PLoS One* (413784), *Proceedings of the National Academy of Sciences of the United States of America* (345531), and *Oncotarget* (345092).

### 3.4 Analysis of authors

A total of 9,779 authors participated in the discourse surrounding this field, encompassing 29 authors who authored single-authored documents and 9,750 authors who contributed to multi-authored documents. Notably, the tally of single-authored documents amounted to 31. The observed average count of co-authors per document stood at 7.3. Of all the authors, Tang JH contributed the most articles (n=24, 1.29% of all articles), followed by Wang Y (n=22, 1.18%) and Zhang J (n=21, 1.13%) (**Supplementary Table S4**). In terms of cited numbers, Tang JH was also the most cited author (580 citations), followed by Zhong SL (500), Pantel K (481), and Zhao JH (481) (**Supplementary Table S5**).

Tang JH had the highest H-index (20), followed by Ochiya T (17), Zhong SL (17), and Zhao JH (15) (**Table 2**). The M-index of Zhang Q (2.167) was the highest, followed by Tang JH (2.000), Zhang F (1.750), and Zhong SL (1.700) (**Supplementary Table S6**). Tang JH exhibited the highest G-index (24), followed by Wang Y (22), Zhang J (21), Ochiya T (20), and Zhang Q (20) (**Table 2**).

**Table 2:** Top 10 contributing authors in the field of exosomes and breast cancer.

Element	H-index	G-index	M-index	TC	NP
Tang JH	20	24	2.00	1585	24
Ochiya T	17	20	1.55	2620	20
Zhong SL	17	17	1.70	1283	17
Zhao JH	15	15	1.50	1224	15
Zhang J	13	21	1.30	969	21
Zhang Q	13	20	2.17	697	20
Wang J	12	18	1.50	772	18
Li J	11	18	1.38	536	18
Wang DD	11	11	1.38	427	11
Wang Y	11	22	1.83	494	22

TC, Total citations; NP, Number of publications.

We examined a cohort of 93 authors who participated in co-authorship collaborations on more than five publications. The highest total link strength belonged to the publications of Tang JH (68), followed by Zhong SL (65), Zhao JH (57), and Yang SJ (40) (**Supplementary Figure S2**).

**Figure 6:**
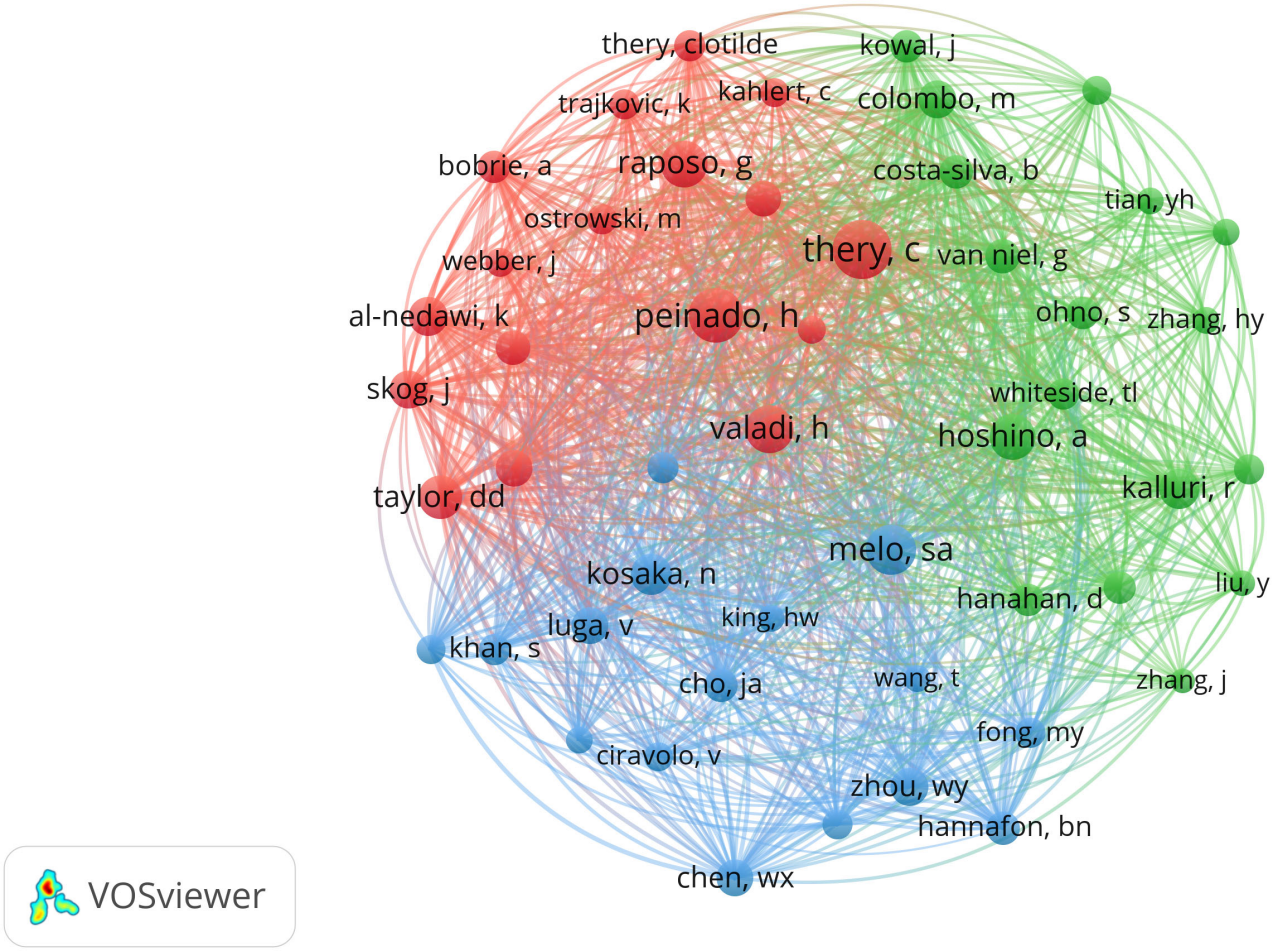
Network map of co-citation of authors whose publications were cited over 100 times.

A co-citation analysis was conducted to assess the authors of cited references, comprising 78 scholars, each with a minimum citation count exceeding 100 times. Among this select group, Thery C stood out with the highest total link strength (5879), followed by Peinado H (5066), Melo Sa (4607), and Valadi H (4250) (**Figure 6**).

### 3.5 Analysis of citations

**Figure 7:**
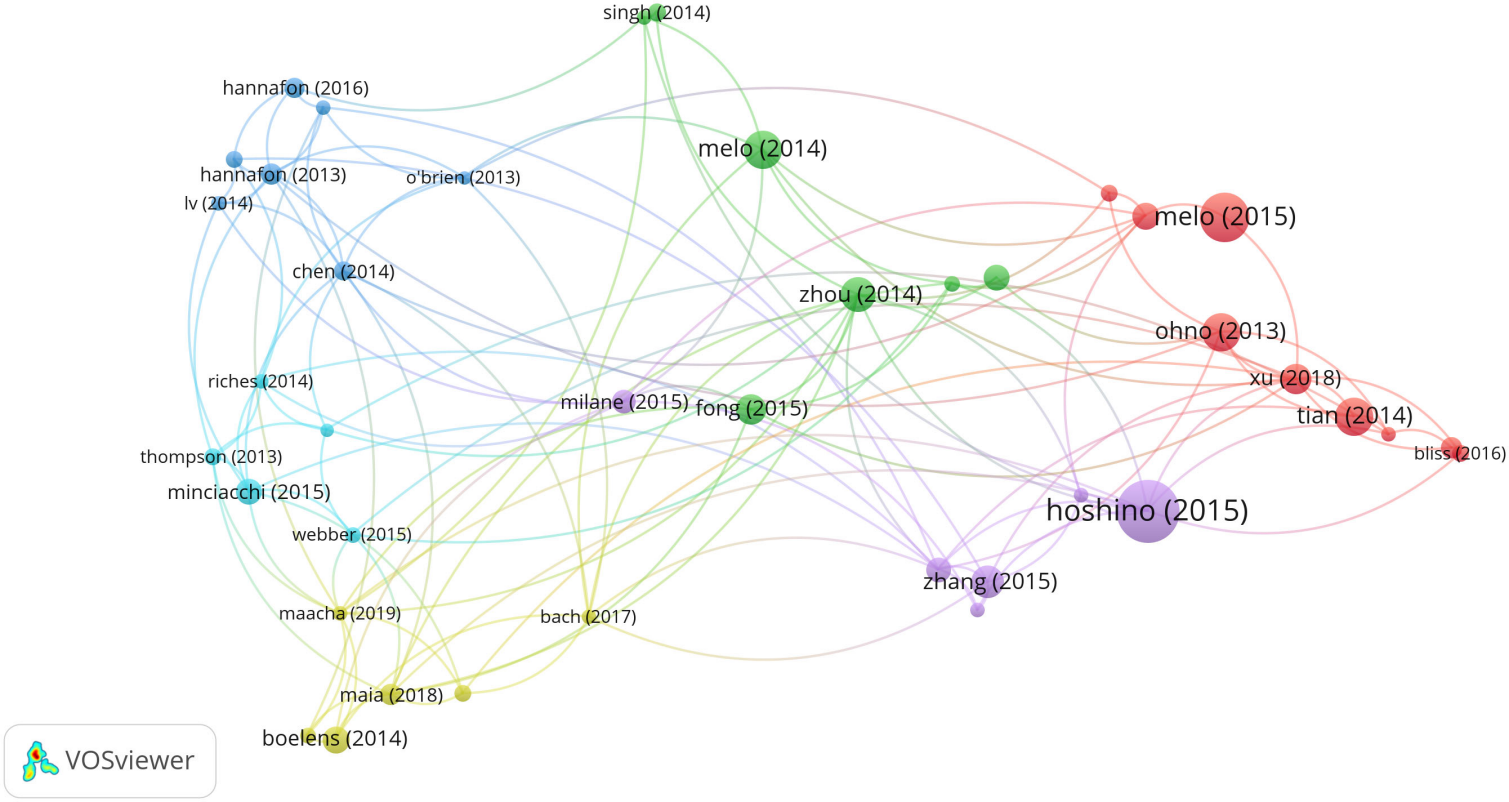
Network map of citation analysis of documents with more than 150 citations.

We analyzed 97 documents that had over 150 citations (**Figure 7**). The top 1 cited document was “Tumour exosome integrins determine organotropic metastasis” (26), with 2730 citations. There were 1688 citations for “Glypican-1 identifies cancer exosomes and detects early pancreatic cancer” (27), followed by “Systemically Injected Exosomes Targeted to EGFR Deliver Antitumor MicroRNA to Breast Cancer Cells” (28), with 1049 citations, and “A doxorubicin delivery platform using engineered natural membrane vesicle exosomes for targeted tumor therapy” (29), with 1047 citations.

### 3.6 Analysis of keywords

**Figure 8:**
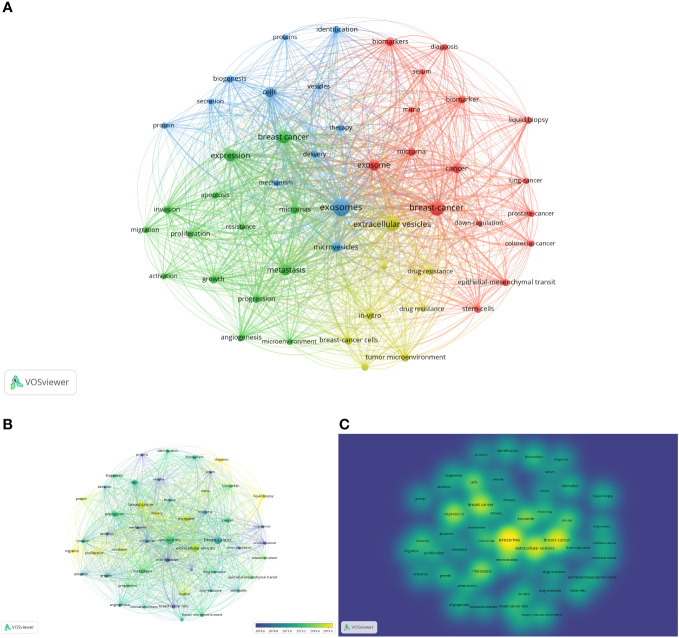
Analysis of keywords. **(A)** Mapping of keywords occurred over 50 times in studies. **(B)** Distribution of keywords according to average publication year (blue: earlier, yellow: later). **(C)** Distribution of keywords according to the mean frequency of appearance (keywords in yellow occurred with the highest frequency).

We meticulously analyzed a compilation of 65 keywords that exhibited occurrences surpassing 50 times (**Figure 8A**). The most frequently occurred word was “exosomes” (occurrences=932), followed by “breast-cancer” (611), “extracellular vesicles” (544), “expression” (373), “metastasis” (314), “cells” (259), “microRNAs” (173), “biomarkers” (158), “tumor microenvironment” (140), “lipid biopsy” (115), “stem-cell” (113), “epithelial-mesenchymal transition” (108), “angiogenesis” (107), and “mesenchymal stem-cells” (102). The main words that occurred over 100 times were selected above. The overlay visualization is color-coded to indicate the average publication year of the identified keywords (**Figure 8B**). Most keywords were published after 2019. Additionally, a density visualization map was adeptly employed to accentuate the identified keywords that exhibit a higher frequency of appearance through deeper coloration (**Figure 8C**).

The thematic map was used to analyze the keywords plus (**Supplementary Figure S3**). There were 6 clusters on the map. The first cluster was blue, including “breast-cancer”, “extracellular vesicles”, and “breast cancer cells”, which had high development degrees and high relevance degrees. The second cluster was brown, including “drug-delivery”, “paclitaxel”, and “brain”, which had high development degrees and low relevance degrees. The third cluster was blue, including “circulating tumor-cells”, “metastatic breast-cancer”, and “peripheral-blood”, which also had a low relevance degree and high development degree. The orange cluster whose relevance degree and development degree were low included “prostate-cancer”, “colorectal-cancer”, and “lung-cancer”. The purple cluster, including “exosomes”, “expression”, and “cells”, had high centrality and modest density. Lastly, the green cluster, including “identification”, “biomarkers”, and “serum”, had high centrality and low density. The purple and green clusters, especially the green ones, may be the research trend and main point of this field in the future.

The CiteSpace V tool was employed to illustrate the top 50 keywords exhibiting the most pronounced citation bursts (**Supplementary Figure S4**). With this figure, the burst period of keywords can be presented. Notably, the keyword “membrane vesicle” exhibited the most robust bursts, characterized by a strength value of 12.25, with citation bursts spanning from 2013 to 2017. The keywords, including “target”, “biology”, “suppressor cell”, “molecular mechanism”, “tumor progression”, “inhibitor”, and “model”, were the focus of current studies, which are still active. From the analysis of keywords, we can find that the futural research will focus on the molecular mechanism and clinical application.

## 4 Discussion

Exosomal dynamics have gained immense importance in the global scientific community and beyond. These tiny vesicles, secreted by cells, are now recognized as key players in intercellular communication, with implications in various fields, including cancer research, disease diagnosis, biomarkers, drug delivery, neurodegenerative diseases, and even biotechnology. The growing number of research articles and reviews on exosomes underscores their significance in the global world for several reasons. With the rapid advancements and global collaboration of the articles, a comprehensive understanding of exosomal dynamics ensures effective collaboration and knowledge exchange among researchers from diverse backgrounds.

We undertook a comprehensive scientometric analysis to elucidate the contemporary research landscape concerning exosomes and breast cancer spanning from 2013 to 2022. In the present study, we used Bibliometrix, VOSviewer, and CiteSpace. As of January 11^th^, 2023, we amassed a total of 1,864 articles. The annual growth rate stood at 29.24%, while the production curve indicated that the number of publications would increase steadily, suggesting a persistent interest in this field. The average number of citations per document was 43.83. The most average citation year was 2015. The types of these ten papers were all articles. Half of these ten papers discuss the effect of exosomes on tumor metastasis (26, 30–33). Three of these ten papers delve into the influence of exosomes on breast cancer metastasis. The miRNAs in exosomes promote metastasis by regulating the migration of endothelial cells, inducing vascular permeability and disruption of the barrier functionality inherent to the endothelial monolayer (26, 32, 33). It is worth mentioning that the most locally cited document, “Tumour exosome integrins determine organotropic metastasis”, just focuses on this topic, which is predominantly on the integrins in exosomes rather in miRNAs. The article also indicates that exosomes redirect metastatic distribution by preparing premetastatic niches, with integrins offering the potential to prognosticate likely sites of metastasis (26). Two of them illustrate that certain exosomes will make cells produce or spread resistance capacity (34, 35). Another pair of articles underscore that plasm exosome microRNAs have the prospective utility as diagnostic biomarkers for breast cancer (27, 36). Moreover, two of them focus on targeted therapy of breast cancer via delivering the drugs by certain exosomes that can bind to certain cancer tissues (28, 29). The remaining document discerns the capacity of exosomes sourced from breast cancer patients to incite epithelial cell carcinogenesis, which also supports the point that exosomes can apply as biomarkers and therapy means (37). In light of these findings, it becomes evident that exosomes hold significant promise in the context of breast cancer research. The emergent research trajectory and focal interests appear to be steering towards the exploration of exosome potential and strategies for their clinical implementation in the realm of breast cancer in recent times.

China was the most productive country. The USA, on the other hand, emerged as the most cited country, claimed the highest total link strength, and ranked second in terms of the number of publications, showcasing significant impact in this domain. The contributions of some developing countries, such as Iran (ranked third in the published number), are also noteworthy.

*Oncotarget* was once the most productive journal in this field from 2016 to 2020. However, the *International Journal of Molecular Sciences and Cancer* went beyond *Oncotarget* after 2021. Besides, *Scientific Reports*, *Frontiers in Oncology*, and *Frontiers in Cell and Developmental Biology* kept a steady trend of growth, which means the significance of molecular science, oncology, and biology.

Tang JH (n=24) was the most productive author and the most cited author, who also exhibited the highest H-index, G-index, and total link strength. In the first year, 2014, he published four related documents. And 11 of his articles involved the drug resistance of cancer.

We can obtain the information from keyword analysis that molecular mechanism was the main point recently, and clinical application is getting more attention. The trend from the broad role of exosomes to specific pathways to breast cancer was revealed by the top 50 keywords with the most robust citation bursts. This shift signifies a gradual progression towards more in-depth and practical investigations. Nonetheless, debates persist regarding the fundamental mechanisms of exosomes, exemplified by contrasting viewpoints on their involvement in the preparation of pre-metastatic niches and their potential role in the reawakening of dormant niches (28). We analyzed keyword plus. The emerging themes included “identification”, “biomarkers”, and “serum”, which needs further research. The keywords with strong citation burst, “suppressor cell sirna”, “molecular mechanism”, and “target” may be the hotpots in the next few years.

The basic themes (“identification”, “biomarkers”, “serum”) all refer to the diagnosis value of exosomes in breast cancer. The new detection method of breast cancer is needed due to the limitation of traditional methods, such as mammography and ultrasonography (38–40). Mammography may fail to detect 10% to 30% of breast cancers (38). Exosomes can be isolated easily and efficiently, suitable as diagnostic biomarkers (41). However, cancer-specific exosomes are difficult to be separated. The current separation methods are complex and not suitable for clinical application. Few articles refer to the clinical method (42, 43). Thus, the ideal separation method may be pivotal to advancing exosome research and their eventual clinical application.

The conspicuous inclusion of the keyword “target” within the top 50 keywords characterized by robust citation bursts is noteworthy. The sphere of targeted therapy for breast cancer has lately undergone rapid advancement. The therapy mainly focuses on two aspects: foremost, impeding the targeted release of cancer-derived exosomes, and secondly, harnessing exosomes as proficient vehicles for drug delivery (44–47). The crux of the initial strategy lies in preserving normal cellular function while effectively curtailing the release of these exosomes. The other aspect also has challenges. The exosomes to deliver drugs must not be contaminated. Furthermore, the intricate methodology governing drug loading warrants further meticulous investigation. Additionally, the route of administration assumes critical significance, attributing to both the heightened immune system response subsequent to exosome administration and the rapid exosomal clearance (47, 48). Indeed, the foremost envisaged application of exosomes in the context of breast cancer revolves around their utility as drug carriers. Exosomes are regarded as efficient and secure vehicles for drug delivery due to their pronounced specificity and minimal immunogenicity (8). An illustrative instance of this principle is exemplified by research wherein exosomes derived from both HEK293 and MCF‐7 cells have been empirically demonstrated to induce anticancer attributes by means of HER2 gene silencing in recipient cells (49).

Furthermore, we have identified previous bibliometric investigations focusing on exosomes; however, our scrutiny unveiled a dearth of analyses specific to their relevance in breast cancer. Notably, a singular article titled “Advances in Breast Cancer Management and Extracellular Vesicle Research, a Bibliometric Analysis” conducted a bibliometric appraisal of related literature spanning from 1998 to 2021 (50). In my assessment, this article stands as a commendable resource for those seeking foundational insights into the inception of this research domain, owing to its extensive temporal coverage. It has notably elucidated the intricate interrelationships among pivotal keywords. Nevertheless, the trajectory of this field has exhibited marked acceleration subsequent to 2021. When juxtaposed with this article, our analysis possesses the advantage of incorporating more recent literature, thereby affording a glimpse into the prevailing research landscape. Furthermore, we have diligently aggregated data from a diverse array of databases, as opposed to relying solely on a solitary source, thereby ensuring a more comprehensive inclusion of seminal contributions. Distinguishing our bibliometric analysis is its capacity to deliver a visually compelling representation within the results section, serving as an invaluable resource for discerning prevailing research trends.

The study we conducted had some limitations. First, the database we used was mainly the WoS database without data from PubMed, Scopus, and other databases. Obviously, the WoS database cannot cover all journals, so the publications in this field we retrieved were not complete. Nonetheless, it is imperative to highlight that WoS stands as the most extensively utilized literature database in scientometric analyses. Additionally, a substantial proportion of bibliometric software is tailored to align with the WoS format. Second, we chose only English articles, which may neglect articles from other languages. Third, certain aspects of the articles, such as the country and category of the journal, proved challenging for the software to analyze fully. In the co-citation analysis of cited authors, only the first author’s information was captured. And the magnum opus of an author cannot be provided. However, we solved this problem by manually searching. It is pertinent to acknowledge that while we have taken measures to mitigate these limitations, they still merit consideration when interpreting the study’s outcomes.

## 5 Conclusion

The contemporary years have borne witness to a rapid surge in research and exploration pertaining to the multifarious role and prospective applications of exosomes within the domain of breast cancer. Within this context, we present a meticulous scientometric analysis of scholarly articles within this domain. This investigation unravels a comprehensive panorama encompassing global annual publication output, relevant countries, organizations, journals, authors, and keywords, illuminating collaborative networks and key seminal works. Moreover, the important documents were provided and generalized. Our scrutiny underscores an intriguing trajectory, where research focus has progressively pivoted from the overarching exosomal landscape towards intricate pathways exclusive to breast cancer, ultimately culminating in the clinical deployment of exosomes for therapeutic intervention. Researchers are committed to finding specific pathways that can be used to apply exosomes to biomarkers and therapy for breast cancer. Besides, some fundamental mechanism of exosomes acting on the breast is still unclear, which may promote the clinical application process. The basic themes, including “identification”, “biomarkers”, and “serum”, are worthy of further study. The keywords with strong citation burst, “suppressor cell sirna”, “molecular mechanism”, and “target”, maybe the hotpots in the next few years. As the burden of breast cancer continues to cast a formidable shadow, the burgeoning promise of exosomes in sculpting the breast cancer paradigm warrants meticulous scrutiny and zealous vigilance.

## Data availability statement

The original contributions presented in the study are included in the article/**Supplementary Material**. Further inquiries can be directed to the corresponding authors.

## Author contributions

JXW: Conceptualization, Writing – original draft. DZ: Visualization, Writing – review and editing. HW: Writing – review and editing. ZZ: Writing – review and editing. QY: Writing – review and editing. JYW: Writing – review and editing. HT: Writing – review and editing. ZJ: Software, Writing – review and editing. LC: Software, Writing – review and editing. JC: Software, Writing – review and editing. YC: Supervision, Writing – review and editing. ZL: Supervision, Writing – review and editing.
