# Supplementary material for: Trends in research of exosomes associated with breast cancer over the past decade: a scientometric analysis
Source: Front Oncol. 2023 Oct 3;13:1273555. doi: 10.3389/fonc.2023.1273555 (PMC10579892; doi:10.3389/fonc.2023.1273555)
Supplement: Supplementary file 1 [file DataSheet_1.docx]

Supplementary Material

**Supplementary Table 1 |** Top 10 countries with the largest number of citations.

| **Country** | **TC** | **Average Article Citations** |
| --- | --- | --- |
| USA | 26955 | 78.10 |
| China | 21342 | 33.10 |
| Japan | 3888 | 63.70 |
| Italy | 3375 | 34.80 |
| Australia | 3168 | 81.20 |
| Korea | 3111 | 43.20 |
| Germany | 2760 | 37.80 |
| Iran | 1935 | 24.20 |
| Canada | 1363 | 42.60 |
| Belgium | 1317 | 101.30 |

Notes: TC=Total citations.

**Supplementary Table 2 |** Top 10 organizations with the largest number of publications.

| **Affiliation** | **Articles** |
| --- | --- |
| Nanjing Medical University | 201 |
| Fudan University | 88 |
| University of Texas MD Anderson Cancer Center | 77 |
| Shahid Beheshti University Medical Sciences | 57 |
| Zhengzhou University | 50 |
| Southern Medical University | 46 |
| China Medical University | 45 |
| Taipei Medical University | 44 |
| University of Alabama at Birmingham | 44 |
| National University of Singapore | 43 |

**Supplementary Table 3 |** Top 10 journals with the largest number of publications.

| **Sources** | **Articles** |
| --- | --- |
| International Journal of Molecular Sciences | 70 |
| Cancers | 64 |
| Frontiers in Oncology | 46 |
| Scientific Reports | 44 |
| Oncotarget | 42 |
| Frontiers in Cell and Developmental Biology | 24 |
| PLoS One | 24 |
| Cell Death & Disease | 23 |
| Cells | 22 |
| Journal of Extracellular Vesicles | 22 |

**Supplementary Table 4 |** Number of publications from the top 10 authors.

| **Authors** | **Articles** | **Articles Fractionalized** |
| --- | --- | --- |
| Tang JH | 24 | 3.03 |
| Wang Y | 22 | 3.01 |
| Zhang J | 21 | 2.93 |
| Chen Y | 20 | 2.27 |
| Ochiya T | 20 | 3.75 |
| Zhang Q | 20 | 2.56 |
| Li J | 18 | 2.08 |
| Liu Y | 18 | 2.03 |
| Wang J | 18 | 1.96 |
| Zhang Y | 18 | 2.09 |

**Supplementary Table 5 |** Top 10 authors with the largest citations.

| **Author** | **Local Citations** |
| --- | --- |
| Tang JH | 580 |
| Zhong SL | 500 |
| Pantel K | 481 |
| Zhao JH | 481 |
| Muller V | 457 |
| Minn AJ | 439 |
| Kalluri R | 420 |
| Liu L | 404 |
| Chen WX | 395 |
| Wang SE | 393 |

**Supplementary Table 6 |** The M-index of publications from different authors.

| **Element** | **M-index** |
| --- | --- |
| Zhang Q | 2.167 |
| Tang JH | 2.000 |
| Zhang F | 1.750 |
| Zhong SL | 1.700 |
| Zhao JH | 1.500 |
| Wang J | 1.500 |
| Yang Y | 1.500 |
| Li J | 1.375 |
| Wang DD | 1.375 |
| Liu Q | 1.333 |


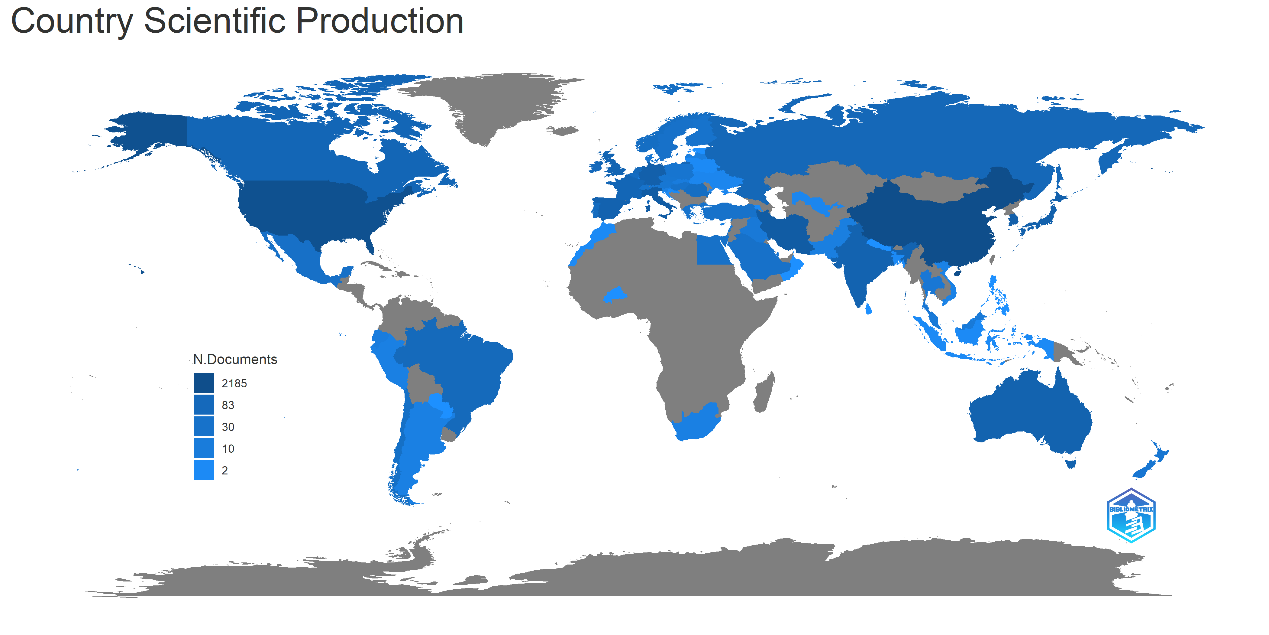


**Supplementary Figure 1 |** World map showing the distribution of countries in this field.


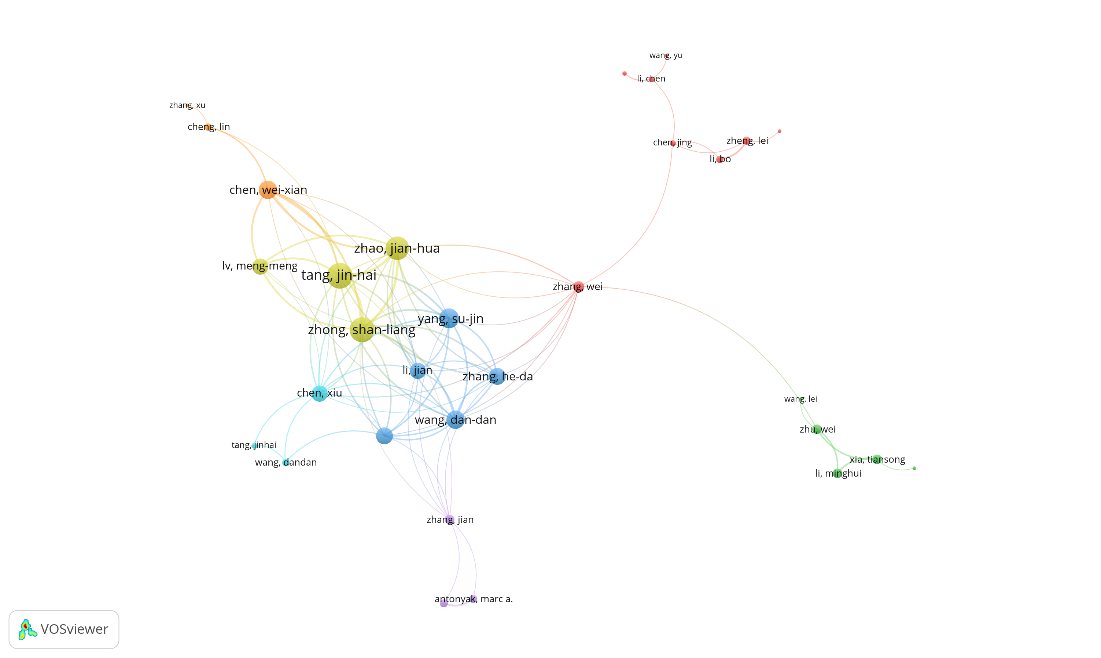


**Supplementary Figure 2 |** Network map of co-authorship between authors with more than five publications.


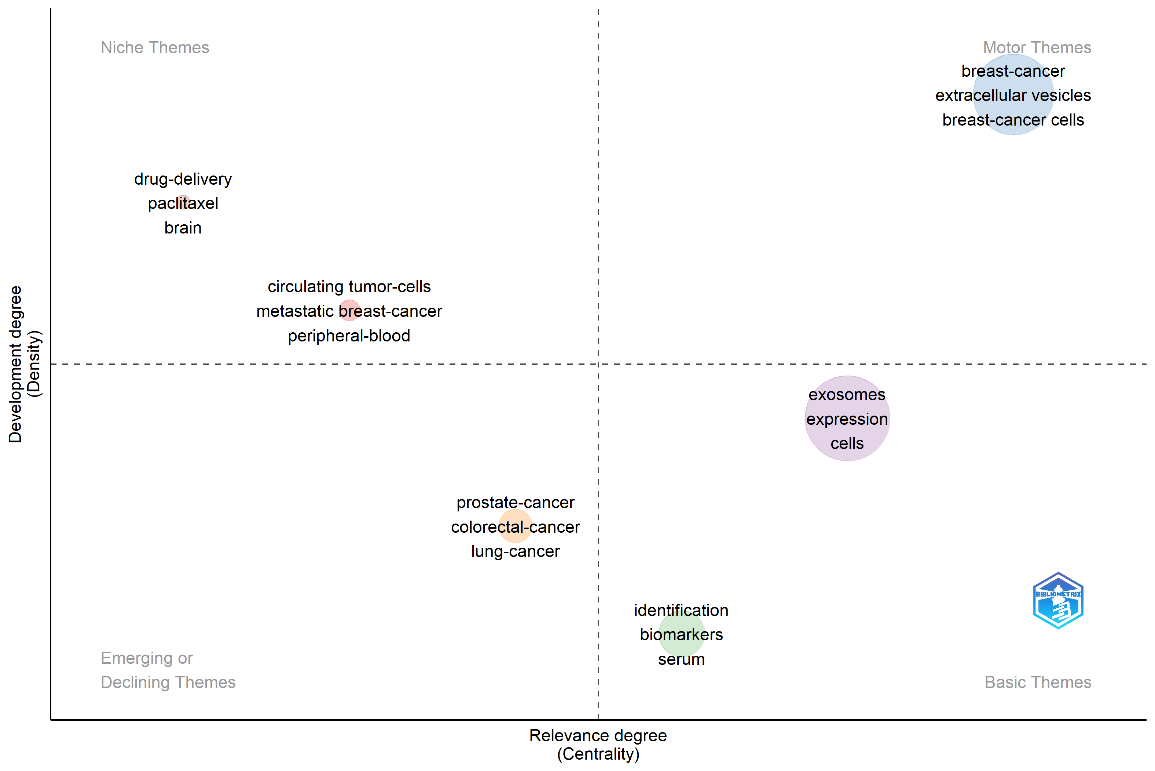


**Supplementary Figure 3 |** Thematic map of keyword plus (different colors mean different clusters).


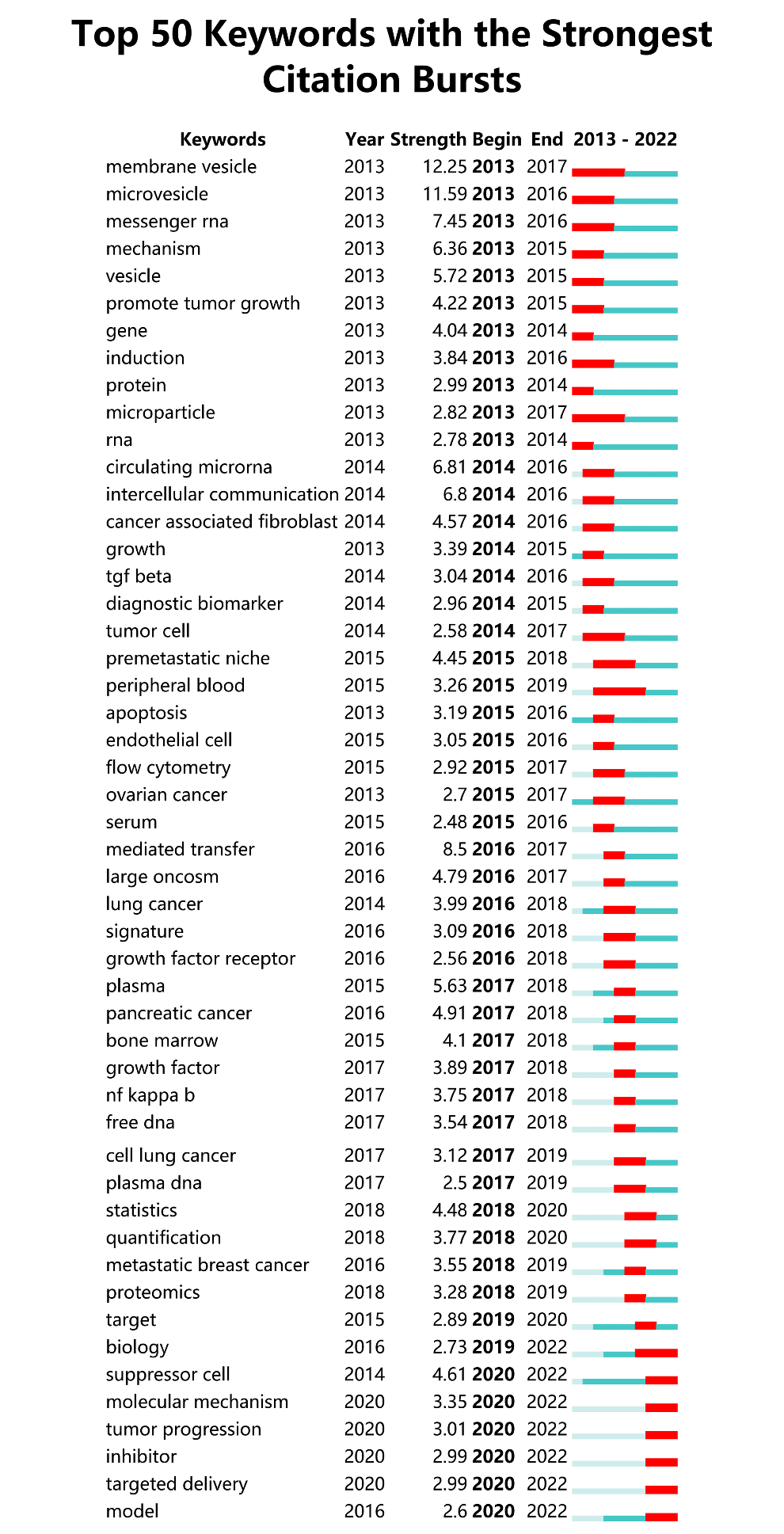


**Supplementary Figure 4 |** Top 50 keywords with the strongest citation bursts.
